# A Blended Electronic Illness Management and Recovery Program for People With Severe Mental Illness: Qualitative Process Evaluation Alongside a Randomized Controlled Trial

**DOI:** 10.2196/20860

**Published:** 2021-01-20

**Authors:** Titus A A Beentjes, Betsie G I van Gaal, Hester Vermeulen, Maria W G Nijhuis-van der Sanden, Peter J J Goossens

**Affiliations:** 1 Radboud Institute for Health Sciences Radboud University Medical Center IQ healthcare Nijmegen Netherlands; 2 Dimence Group Mental Health Care Centre Deventer Netherlands; 3 Saxion University of Applied Science Center for Nursing Research Deventer Netherlands; 4 Faculty of Health and Social Studies HAN University of Applied Sciences Nijmegen Netherlands; 5 Department of Public Health and Primary Care, Faculty of Medicine and Health Sciences University Centre for Nursing and Midwifery Ghent University Ghent Belgium

**Keywords:** mental health recovery, self-management, telemedicine, mental health services, qualitative research

## Abstract

**Background:**

We conducted a trial to test the electronic Illness Management and Recovery (e-IMR) intervention to provide conclusions on the potential efficacy of eHealth for people with severe mental illness (SMI). In the e-IMR intervention, we used the standard IMR program content and methodology and combined face-to-face sessions with internet-based strategies on the constructed e-IMR internet platform. During the trial, the e-IMR platform was sparsely used.

**Objective:**

This study aimed to evaluate the added value of the e-IMR intervention and the barriers and facilitators that can explain the low use of the e-IMR platform.

**Methods:**

This process evaluation was designed alongside a multicenter, cluster randomized controlled trial. In this study, we included all available participants and trainers from the intervention arm of the trial. Baseline characteristics were used to compare users with nonusers. Qualitative data were gathered at the end of the semistructured interviews. Using theoretical thematic analyses, the data were analyzed deductively using a pre-existing coding frame.

**Results:**

Out of 41 eligible participants and 14 trainers, 27 participants and 11 trainers were interviewed. Of the 27 participants, 10 were identified as users. eHealth components that had added value were the persuasive nature of the goal-tracking sheets, monitoring, and the peer testimonials, which had the potential to enhance group discussions and disclosure by participants. The low use of the e-IMR platform was influenced by the inflexibility of the platform, the lack of information technology (IT) resources, the group context, participants’ low computer skills and disabilities, and the hesitant eHealth attitude of the trainers.

**Conclusions:**

The extent of eHealth readiness and correlations with vulnerabilities in persons with SMI need further investigation. This study shows that flexible options were needed for the use of e-IMR components and that options should be provided only in response to a participant’s need. Use of the e-IMR intervention in the future is preconditioned by checking the available IT resources (such as tablets for participants) providing computer or internet guidance to participants outside the group sessions, evaluating the eHealth attitude and skills of trainers, and tailoring eHealth training to increase the skills of future e-IMR trainers.

**Trial Registration:**

Netherlands Trial Register NTR4772; https://www.trialregister.nl/trial/4621

**International Registered Report Identifier (IRRID):**

RR2-10.1186/s12913-016-1267-z

## Introduction

### Background

In mental health care, eHealth is expected to have great potential to increase access to care while being economically and socially efficient [1]. eHealth can be defined as making use of information technology (IT). In meta-analyses, eHealth interventions for persons with depressive and anxiety disorders are accepted and proven to be effective [2]. eHealth is also used for persons with severe mental illness (SMI). Persons with SMI are diagnosed with a psychiatric disorder that causes, and is because of, serious impairments in social and occupational functioning that lasts longer than at least a couple of years and necessitates coordinated multidisciplinary care [3]. eHealth for persons with SMI is used in a wide range of interventions, such as self-management, relapse prevention, promoting adherence to medications and/or treatment, psychoeducation, supporting recovery, and promoting health and wellness and symptom monitoring [4]. eHealth interventions for people with SMI are accepted and feasible [4], and they have potential to deliver effective education [5]. Unfortunately, conclusions on their effectiveness cannot be drawn [4,6]. A number of difficulties and barriers have been addressed concerning eHealth for persons with SMI (eg, cognitive impairments, lower IT experience [7]), which may explain the high attrition rates [8]. Blending face-to-face contact with eHealth is supposed to increase the therapeutic relationship and prevent attrition [9].

To contribute to consumer-oriented development and delivery of self-management electronic support programs, we developed and tested a blended version of the standardized, curriculum-based Illness Management and Recovery (IMR) program for people with SMI [10,11]. The standard IMR program provides information and teaches the skills necessary for managing an SMI effectively and working toward achieving personal recovery goals [12]. In accordance with the intervention mapping (IM) protocol [13] and in collaboration with target group members, we developed the e-IMR intervention to evaluate whether persons with SMI could benefit more from the IMR when making use of eHealth strategies in combination with face-to-face sessions [11]. On the e-IMR internet platform, the IMR curriculum was integrated, and we blended the use of this platform with face-to-face, group-wise delivery of the standard IMR program [11]. To evaluate the effectiveness of the e-IMR intervention compared with the standard IMR program, we conducted a multicenter, cluster randomized controlled trial [10,11].

The most striking finding of the trial was the low use of the e-IMR platform [10]; therefore, we could not conclude the effectiveness of the e-IMR intervention. Sieverink et al [14] reported that many eHealth evaluations show no or limited positive effects, which is strongly related to not using technologies in the desired way. Ben-Zeev et al [6] advised that the development of eHealth interventions for people with SMI must be coupled with examining the barriers and possible solutions. In addition, the IM protocol advises testing the effectiveness of an intervention and conducting a process evaluation to understand why an intervention did or did not work [13]. Therefore, we conducted this process evaluation alongside a randomized controlled trial to gain insights that will ultimately help to make adjustments to facilitate proper use of the e-IMR intervention specifically or of eHealth for people with SMI in general.

### Objectives

This study aimed to identify the added value of the e-IMR intervention and the barriers and facilitators that can explain the low use of the e-IMR platform.

## Methods

### Study Design

We conducted a theoretical thematic analysis [15] alongside the trial. This qualitative method makes use of a pre-existing coding frame and provides a detailed analysis of the data [15]. Data were derived from semistructured interviews with participants and trainers held at the end point of the trial. This trial was registered in the Netherlands Trial Register (NL4621). We used the framework of Grol and Wensing [16-19], which frames the factors that potentially influence the effect of an intervention (Multimedia Appendix 1) [17]. Therefore, we focused on the e-IMR intervention itself and its implementation, the trial participants and their social context, the IMR trainers who provided the intervention, and their organizational context.

### Study Population

**Figure 1:**
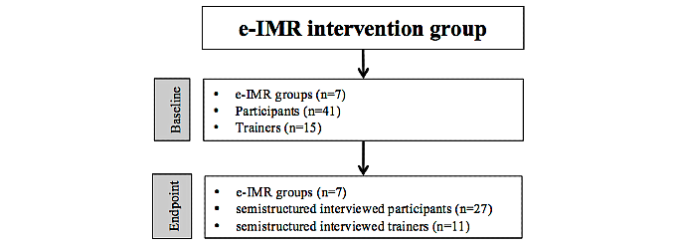
Study flow diagram. e-IMR: electronic Illness Management and Recovery.

In this study, we included all available participants and IMR trainers from the intervention arm of the e-IMR trial [10] (Figure 1). Information about inclusion, exclusion, and eligibility criteria and the effect of the e-IMR trial can be found elsewhere [10]. Participants in the intervention arm of the trial who completed at least the first module on the e-IMR platform or had logged into the e-IMR platform at least five times were defined as *users*. Nonusers either did not use the e-IMR platform or used it less than 5 times. Users were regarded as having had the opportunity to benefit from the e-IMR intervention and to reflect on it. The trainers of the group-wise–delivered e-IMR intervention were psychiatric nurses and peer professionals. A peer professional is a person with a lived experience of a mental illness, educated, and trained to become a professional capable of transferring knowledge and counseling other persons with a mental illness.

### The e-IMR Intervention

The e-IMR intervention started with a *welcome page* explaining the use of the e-IMR platform and leading participants to the 11 modules. On the e-IMR platform, participants could fill in e-versions of the forms in the standard IMR, such as goal-tracking sheets, problem-solving sheets, sheets for tracking successful coping strategies, and a symptom-monitoring page. In addition, the e-IMR platform contained illustrative videos showing peer testimonials to encourage participants to talk more freely about themselves and to take steps in their recovery process. Further detailed information about the e-IMR intervention is shown in Multimedia Appendix 2.

### Implementation of the e-IMR Platform

The e-IMR platform was introduced to the trainers and participants by the first author at the second group session. Participants were invited to use the e-IMR platform but were not obliged to use it at home because of the possible lack of resources. The trainers were educated on how to support participants in the use of the e-IMR platform, how to install it on a computer in the session room, and how to use it during the sessions. The registration forms on successful coping strategies and the symptom-monitoring page were introduced after the second module on *practical facts about mental illnesses*. Weekly emails with a link to the e-IMR platform led the participants directly to the symptom-monitoring page. After finishing any module, one of the trainers provided feedback to the participants via the platform and guided the participants to the next module. Further detailed information about the implementation of the e-IMR intervention is presented in Multimedia Appendix 2.

Halfway through the trial, we discussed the low use of the platform with the trainers and asked them to reintroduce the platform in the sessions and to motivate and guide the participants to use it at home to get as much experience with it as possible. With regard to this request and in addition to the original implementation strategy, in 4 out of the 7 groups, extra e-IMR lessons were organized outside the current IMR sessions (Multimedia Appendix 2).

### Data Collection

Data were collected between January 2015 and October 2016. Three types of data were gathered: participants’ characteristics, log data of the use of the e-IMR platform, and qualitative data from semistructured interviews at the end point of the trial.

At baseline, the following data on participants’ characteristics were gathered: age, gender, diagnostic classification according to the *Diagnostic and Statistical Manual of Mental Disorders* (4th edition) [20], physical comorbidities, treatment history, cultural background, socioeconomic status, highest education, computer and internet availability and use, computer literacy, perceived computer skills, and the need for guidance when using a computer or the internet. The last 2 items were scored on a 5-point Likert scale, with the answer options of *strongly disagree* (1) to *strongly agree* (5). In addition, the following data from trainers were collected at baseline and used for this study: age, gender, profession, highest education, years of experience in mental health, and eHealth experience.

Log data about the actual use of the e-IMR intervention were derived from the e-IMR platform. These data were used to identify *users* and *nonusers*.

We conducted semistructured interviews at the end point of the trial with all available participants and trainers. After the halfway discussions with the trainers about the low use of the e-IMR platform, we discussed the potential influential factors and adapted the framework of Grol and Wensing [17]. Within each factor, we formulated a number of relevant determinants and accordingly set up the interview questions (Multimedia Appendix 1). The framework and questions were used as the interview topic list in semistructured interviews at the end point. The first author (TB) and research assistants performed the interviews with participants at their preferred location. The first author conducted interviews with the trainers. All semistructured interviews were audio recorded and transcribed verbatim. The transcripts were uploaded in Microsoft Excel (R).

### Data Analyses

Descriptive statistics were used to present the outcomes for the groups of users and nonusers. Chi-square and Student *t* tests were carried out to compare the baseline characteristics and IT attitudes of the groups of users and nonusers. Quantitative data from the structured interviews at baseline and end point were analyzed using the Statistical Package for the Social Sciences (R) version 23 [21].

Data from the transcripts of the semistructured interviews were analyzed deductively using theoretical thematic analysis [15]. We used the following 7 steps:

1. All 3 authors (TB, BG, and PG) independently read and reread the transcripts from the participants and from the trainers for one e-IMR group and identified meaningful statements.
2. All 3 authors grouped the statements into the categories of the modified coding frame of Grol and Wensing [17].
3. All 3 authors triangulated their analyses thoroughly until consensus was reached, which means that discussions lasted until all agreed without any doubts.
4. The first author completed the analyses of the subsequent e-IMR groups according to the first 2 steps.
5. The first author formulated a description of the findings within each determinant and added verbatim examples.
6. All 3 authors discussed the description of the findings thoroughly until consensus was reached.
7. Finally, a composite description of the experiences and the use of the e-IMR intervention was written and discussed with all authors.

## Results

### Characteristics of Participants and Trainers

From the 7 groups, baseline characteristics were collected from 41 participants and 15 trainers (Multimedia Appendix 3). The mean age of the participants at baseline was 46.9 years (SD 11.6; n=41) and the majority had minimal income. The mean age of the trainers at baseline was 46.7 years (SD 8.8; n=15). In total, 9 trainers were psychiatric nurses and 5 were peer professionals. Of the 41 participants, 14 (34%) were identified as e-IMR users. The groups of users and nonusers only differed significantly according to gender (*P*<.042), with more men being nonusers.

### Process Evaluation

At the end point, 27 participants (10 of whom were users) and 11 trainers were available to be interviewed (Figure 1 and Multimedia Appendix 3). A total of 14 participants (4 of whom were users) were not interviewed because they were too burdened by being interviewed. From all the e-IMR groups, at least one trainer was interviewed; 4 trainers were unavailable because of busy work schedules. In the following sections, the findings are reported according to the framework (Multimedia Appendix 1). In our findings, we used the terms *users* or *nonusers* to make it clear that among the participants, only users or nonusers reported the mentioned statement. We used the term *participants* when both users and nonusers reported the statement. The following section details the findings for the e-IMR intervention and its implementation, the trial participants and their social context, and the IMR trainers who provided the intervention and their organizational context. The determinants for these factors are illustrated by using quotes from participants coded with a P followed by 4 digits and either U or N (standing for *user* or *nonuser*, respectively) and by quotes from trainers coded with a T followed by 5 digits and either Pe or Nu (standing for *peer professional* or *psychiatric nurse*, respectively).

### The e-IMR Intervention and Its Implementation

Regarding the e-IMR intervention, the following determinants are described: added value, accessibility, implementation fidelity, and feasibility.

#### Added Value

Users and trainers reported that the components of the e-IMR intervention had added value. One user stated that because of the platform, the standard IMR curriculum was easier to understand. Explanations on relevant subjects in the different modules, for instance, about symptoms, were easy to find using the buttons on the platform. A trainer mentioned that the time-consuming search in the textbook was no longer necessary. In 4 out of the 7 groups, peer testimonial videos were shown during the group sessions. Watching these videos was of great value to trainers and participants, enhancing discussions and disclosure. Participants found the peer testimonials very interesting and experienced recognition:

Yes, those videos … I liked them. Watching them was the first we did, and it became easier to talk about the subject.

T31002Pe

However, sometimes the participants felt fearful when reminded of their own psychotic experiences.

Trainers and users reported the added value of the repetitive character of the goal-tracking sheets on the platform. Users easily tracked and celebrated their achievements. When only the hard copy module was used, the paper goal-tracking sheets were often lost, which hindered the monitoring of achievement over time:

So, your goals appear; that’s not in the book. . . . it’s not possible to drop your focus. You’re reminded of them ...

P1202U

One user reported that the results of the weekly reminders to monitor symptoms led to a more objective interpretation of varying emotions, which increased personal insight. Another user did not benefit from this. A different user thought that the focus on symptoms was too strong, and one peer professional trainer mentioned that he experienced aversion to this assignment because of this focus on symptoms:

In every chapter it appears: How much did symptoms burden you? ... it’s too negative. I know it is meant to be positive .... But, huh [shivering], these symptoms again; f[...] off!

T51003Pe

The users and trainers reported that they did not use the *coping strategies* and *problem-solving sheets*.

#### Accessibility

Most of the participants reported that the eHealth components were not easy to find. Out of 14 users, 6 reported having problems with logging on to the e-IMR platform at home. In 5 of the 7 groups, participants and trainers reported that accessing the platform during the sessions was problematic because of bugs when using certain browsers, problems with accounts, problems with logging in, and not having the appropriate IT resources:

Someone from technical services helped them, but the trainers couldn’t get it running. The enthusiasm in the group to work with it was very low. So they stopped trying, and we worked with the book the rest of the time.

P1106U

#### Implementation Fidelity

Trainers stated that they gave enough attention to motivate participants to use the e-IMR platform. Both trainers and participants reported that because of problems with accessing the platform and the aversive reaction of nonusers, the actual use of the platform during the sessions was low, apart from the peer testimonial videos. Moreover, participants reported that using the e-IMR platform at home was not discussed in later sessions. Some users felt that the trainers did not stimulate them and that they linked this to the fact that the use of the e-IMR platform was not obligatory:

It was like: “It is no obligation, I can do it, but ....” I think that when it got more attention, you’ll be able to see what it’s bringing you.

P1202U

#### Feasibility

The participants and trainers reported the nonfeasibility of working on a computer with a projector and screen during the sessions. They estimated that it would be too time consuming to switch from 1 participant’s account to another. Furthermore, participants could not read their own homework notes when they watched another participant’s account on the projection screen. They thought that the use of a personal laptop or tablet could overcome this problem:

I wondered how a session would go when we do everything in the e-IMR, and nothing on paper. What if someone else is active on the screen, and then I can’t see my own notes? What did I write down at home? I won’t remember, unless we all have a tablet or laptop.

P4202N

Trainers and users reported inflexibility of the platform, such as not being able to amend notes or skip an uninteresting module or change the module order. As the platform was not used adequately during the sessions, participants easily lost synchronicity: doing the e-IMR intervention on the platform at home and during the group sessions became 2 separate things. Nonusers reported that they stopped or did not start using the platform to avoid duplication of effort and to prevent confusion by using 2 ways of working with the IMR:

There are two things ... I was afraid to mix them up ... So, you do double work. You choose either the book or the platform ... not both.

P3104N

### The Participants

Regarding the participants, the following influencing determinants can be described: attitude, compliance, skills and knowledge, and resources.

#### Attitude

With regard to computers, nonusers reported that they postponed the use of computers, were not interested, did not have an affinity, felt that working with computers was impersonal, were too easily overstimulated by the overload of content on a computer screen, experienced a lack of control over what was happening in the computer, and had a preference for tangible paper and face-to-face communication. Some nonusers experienced fear and mistrust in the privacy protection of the e-IMR platform, not wanting to take the risk of others being able to read their notes:

I don’t know where my information goes when I am on the world wide Internet. I need control, always and ever. ... I will get over-stimulated, all those things in my site, they really distract me.

P4207N

#### Compliance

Some users said that they got lost and confused when confronted with the platform’s inflexibility or when they wanted to get through a backlog after a short period of not using the platform. Users missed additional stimuli from trainers to deal with this backlog. Not using or stopping use was related to vulnerability, such as wanting to avoid burdens because of duplication of effort, not feeling well enough, having sensory overload, a lack of concentration, dyslexia, perfectionism, or fear of failure:

Yes, in the group you can talk it out right away; that’s easy ... I did not like doing it on the computer. I think because of the upcoming emotions ... and being alone here at home, no one to talk with ... It just was too much for me, and I decided to stop using it.

P1207N

In terms of vulnerability, the trainers added that the participants recently experienced psychosis, lived a chaotic life, lacked inquisitiveness and initiative, had low intelligence, or had learning disabilities. Learning new skills was reported to be too difficult when not feeling well. The opposite was also reported—feeling better halfway through the trial and then being able to use the platform:

First I thought: This looks handy; I can do it. I really intended to do so. But I got those mood swings and thought: Let me do it on paper; it’s ... what I am used to do ... and I will do it later when I feel well enough — then I will. But that did not work.

P4103N

#### Skills and Knowledge

At baseline, 15% (6/41) participants reported that they had never used a computer and most participants (27/41, 66%) scored neutrally or agreed that they had good computer skills. At the end point, participants reported not being familiar with computers, being afraid of computer viruses from the internet, not knowing how to log in, and not being able to imagine how computers process their input:

I cannot work on the computer .... I did try to learn, but ... no. Terrible, I might be able in a year or so. Now I really cannot.

P1103U

At baseline, 34% (22/41) of the participants did not agree that they needed guidance in working with computers. Of these participants, 29% (14/41) became users. At the end point, participants with a need for guidance reported reluctance in asking for help. A total of 3 participants became a user halfway through the trial with considerable support from the trainer. One trainer illustrated how a user was helped with working on the e-IMR platform:

Well, I (trainer) was at the computer. She (a user) was sitting next to me, and I asked: “Shall I click here or there?” I typed the text and repeatedly asked: “Is this correct?”

T41001Nu

#### IT Resources

In total, 8 participants (8/41, 20%) reported having no computer but one of them did become a user. Moreover, not having the internet, an email account, or finances to afford these resources was reported. Most (31/41, 76%) of the participants had minimal income:

No, really, I was angry; at that time, I had lost my computer. I did it the old-fashioned way. I was fed up with that d[...] computer....

P3101N

### The Social Context of the Participants

Within the social context of the participants, the following determinants can be described: social support and group effect.

#### Social Support

A female user reported that getting help to use a computer from her partner caused irritation. She preferred the help of someone outside her family. Other participants reported that they had a partner with no computer skills. In total, 3 participants became users after getting help from relatives, friends, or trainers outside the group session:

I’ll tell you, I just met him, and he fixed the necessary update. I did not dare to open it, and that’s over now ....

P1204N

#### Group Effect

In 4 out of the 7 e-IMR groups, the participants decided not to use the e-IMR platform during the sessions. A nonuser decided not to use the e-IMR platform at home because another person in the group (a user) was struggling obsessively with using the e-IMR at home. The users and trainers experienced a negative group attitude toward the e-IMR platform, for instance, when nonusers expressed their irritation when the e-IMR platform was discussed during the sessions:

Yes, those participants who were not active on the e-IMR platform were irritated and said: “Why talk about the e-IMR again? ....”

T11003Nu

### Trainers

Regarding trainers, the following determinants can be described: attitude and skills and knowledge.

#### Attitude

Most trainers reported not being computer minded or having a preference for face-to-face contact and tangible paper:

I ’m not that Internet-minded; nor is my colleague. ... My colleague prefers working with these flipcharts.

T42002Pe

The trainers estimated that helping participants with the use of the platform during the sessions would take too much time. They differed on whether offering individual guidance to the participants was part of their job as an IMR trainer. The trainers doubted, and some did not offer lessons on using the e-IMR platform outside the group:

Yes, ... a participant had intentions to start, but had troubles with the computer firewall .... I was wondering, ... what can I do to lower barriers? One option was to install things on her computer, but I considered this was going too far.

T12001Pe

Some trainers reported that they observed vulnerabilities, disabilities, lack of concentration, easy loss of self-esteem, lack of discipline, and struggle with computers in participants. The trainers suggested that participants belonged to a generation with less computer experience and thought that this was influential. Thus, some trainers stated that combining eHealth and SMI is a complete misfit, and they blamed the policy makers for this:

This trend is politically grounded ... this e-mental health, blah, blah. Well, I think people from behind their desk invented this. They do not know what people with SMI go through.

T11003Nu

The trainers reported that working with the e-IMR intervention and motivating participants was an extra, burdensome effort. They felt that working with the e-IMR intervention disturbed the group sessions and that doing the IMR regularly and working with a group were already difficult. The trainers reported cautiousness in opposing the resistance of nonusers to the e-IMR intervention. Their priority was to work with the IMR content and prevent participant attrition from the sessions, and the e-IMR platform became an afterthought:

I think most important in the group is that we go on and follow the book. In fact, working on the e-IMR platform was a sideshow.

T12003Nu

#### Skills and Knowledge

At baseline, one trainer had eHealth experience. Some trainers reported having had enough tools; however, others reported not having heard enough about the e-IMR intervention and the trial. The trainers gave differing reports on whether they had enough skills; some said they did not:

My colleague explained to me how to start the e-IMR platform, but when I am alone, like today, I can’t manage.

T42001Nu

### The Organizational Context of the Trainers

Regarding the organizational context of the trainers, the following determinants can be described: policy, IT resources, and workflow.

#### Policy

The trainers had difficulty logging on to the platform because of a privacy policy in their organization. The internet system of organizations had firewalls to protect the organizations’ IT environment for internet viruses. Owing to this, some websites and email addresses were identified as unsafe and were blocked:

Here [via our intranet], I can’t enter LinkedIn or Dropbox ...you can’t enter hardly anything. ... They’re afraid of viruses.

T31001Nu

#### IT Resources

At the start of the trial, one organization had an IT environment that was compatible with the e-IMR intervention but the other organizations did not. The session rooms often lacked a computer, a soundcard in the computer, Wi-Fi, a projector, and a screen. The trainers sought help from IT help desks in their organizations but could not resolve these problems. Some trainers were creative and determined to find a bypass outside the local IT environment:

S[...], to get the video’s work, the sound card was blocked, but I thought: “I won’t quit trying. I want to show them.” ... In the end, we made it.

T42002Pe

#### Workflow

In the search for another session room, trainers were confronted with overly strict schedules. Another issue was about starting IMR groups and assigning IMR trainers on time. In a number of organizations, IMR groups could only be organized shortly before closing the trial period. The trainers reported that such workflow problems are *business as usual*. To fulfill the participants’ need for guidance with the e-IMR intervention, a number of trainers reported not having enough time in their work schedule:

But this person needs guidance every day. I do not know how to manage that. I don’t have time to do so.

T52001Nu

## Discussion

### Principal Findings

In this study, we evaluated the added value of the e-IMR intervention and the barriers and facilitators that can explain the low use of the platform. The users and trainers had negative and positive experiences with the e-IMR intervention. The added value of the e-IMR intervention consisted of the peer testimonial videos, the persuasive nature of the monitoring page, and the weekly confrontations with their personal recovery goals. There were barriers in the platform’s inflexibility, the infeasible group-wise provision of the intervention, the hesitant attitude toward eHealth of the participants and trainers, the participants’ lack of IT resources, their low skills and knowledge of using the internet, and their being too overwhelmed by symptoms or disabled cognitive functioning, causing problems with using the e-IMR platform.

### Strength and Limitations

The strength of this study is that it included people with low computer use, which enabled us to obtain a broad picture of the added values, barriers, and facilitators. A limitation of this study is that we cannot draw conclusions about the potential feasibility of the e-IMR intervention in individual treatment settings. The e-IMR intervention might work better in individual sessions, as it can be better tuned and tailored to the personal needs of the person with SMI. We estimate that the influence of the group attitude and the e-IMR intervention’s infeasibility in group settings were considerable. Unfortunately, the institutes where IMR is provided individually declined to participate in the trial.

### Comparison With Previous Work

The weekly monitoring page worked out well for some users; however, for others, including a trainer, these reminders were disliked because of a strong focus on symptoms. For users, the weekly confrontations with personal recovery goals and actions worked better than the paper version. The peer testimonial videos were highly appreciated because of their potential to enhance group discussions and the disclosure of the participants. Peer testimonials fulfill the need for peer information and acknowledgment [22]; thus, watching this kind of video can be a pivotal experience that enhances reflection and discussion [23].

Users and trainers were confronted with the platform’s inflexibility when they wanted to emend previous notes, skip uninteresting modules or the monitoring page, and change the order of the modules. Therefore, the next version of the e-IMR intervention should be flexible to fit individual needs. Addressing personalization seems to be a key issue for future eHealth interventions for people with SMI [24]. The group-wise provision of the e-IMR intervention was experienced as infeasible because it was too time consuming to switch between the accounts of participants and the fact that participants were not able to look at their own notes. In addition, because of their e-IMR–averse attitude, participants chose not to use the e-IMR platform during the sessions. Unintentionally, the e-IMR platform and the face-to-face IMR session became 2 separate things. To overcome this group barrier, providing a tablet to participants was a widely heard suggestion. Providing devices to persons with SMI is known to support engagement in e-interventions [23]. This may also overcome the lack of IT resources in persons with SMI, which in our study group was present in 20% (n=41) of participants, comparable with the percentages found by Thomas et al [25]. A lack of IT resources was also present in the participating institutes. Future e-IMR–providing institutes need an open IT environment, open soundcards, strong computers, Wi-Fi for multiple tablets, an available projector plus screen, and a help desk. Technological resources are necessary to facilitate eHealth interventions [24].

The lack of computer skills and the preference for tangible paper and face-to-face communication of the participants can also be seen as barriers. Belonging to a generation with less computer experience might be an influence because low computer literacy is associated with higher age [26]. Similar to Williams et al [23], we identified log-in problems and problems with finding e-components on the platform, which contributed to the low use of the e-IMR platform. The participants in our study thought that their problems with learning and using the e-IMR platform were because of being too overwhelmed by their symptoms or a disability in their cognitive functioning. Executive functions, working memory, and sustained attention play an important role in using websites [7], and these functions are also highly associated with psychiatric illnesses [27,28]. To gain a clear picture of the correlation between the psychiatric health status of persons with SMI and their eHealth readiness, more research is necessary. Taking our findings regarding participants’ attitudes, compliance, and skills and knowledge, we concluded that most participants in our study were not yet ready to engage with eHealth. Berry et al [29] drew a comparable conclusion that persons with SMI have relatively low interest in and willingness to engage with eHealth interventions. We also concluded that there is a need for guidance in persons with SMI. Implementation of the renewed e-IMR intervention must coincide with an eHealth support intervention for participants. Successful use of internet-based interventions for persons with SMI is facilitated by training, support, and encouragement [23].

The eHealth attitudes, skills, and knowledge of trainers toward the e-IMR intervention are also barriers. Their hesitant attitude toward eHealth is based on their preference for tangible paper and face-to-face contact and their own low computer skills. The trainers in this study stopped promoting the e-IMR intervention so they would not burden the participants and avoid causing the participants to withdraw from the sessions. Identifying with the participants’ struggles and vulnerabilities might also have influenced the process. To illustrate, some of the trainers questioned the appropriateness of eHealth for people with SMI, blaming the policy makers. Dutch mental health nurses indicate that eHealth is not in line with the educational level, cultural background, or digital skills of mental health patients [30]. Williams et al [23] suggested a paternalistic attitude when workers determine the suitability of using eHealth interventions for persons with SMI. In this study, in 4 out of the 7 groups, trainers strived creatively to find solutions for showing the peer testimonial videos and organizing e-IMR lessons outside the group sessions. Owing to this effort, 3 of the non–computer-minded participants became *users*. Worker engagement is essential to the successful implementation of eHealth for persons with SMI [23]. Before implementing the renewed e-IMR intervention, it might be necessary to teach trainers how to use eHealth, become experienced, and resolve their hesitancy.

Despite our findings, the development of internet-based interventions is ongoing in our increasingly digitalizing society and health care. Strand et al [24] stated that the internet can play a transitional role in recovery-oriented practices, and Williams et al [23] identified its potential to elicit the personal values of persons with SMI and their treatment preferences. These promising statements make further development of the e-IMR intervention worthwhile.

### Conclusions

The eHealth components of the e-IMR intervention that have added value are the persuasive nature of using goal-tracking sheets and monitoring and the potential of the peer testimonial videos to enhance group discussions and the disclosure of the participants. The low use of the e-IMR platform was influenced by its inflexibility, lack of IT resources, group context, lack of computer skills of the participants and their disabilities, and the hesitant eHealth attitude of the trainers. The extent of eHealth readiness and the correlations with vulnerabilities in persons with SMI need to be investigated further. Providing the e-IMR intervention in the future is preconditioned by the flexible use of components in response to a participant’s needs, checking the available IT resources in institutions, providing tablets to participants in group settings, providing computer or internet guidance to participants outside the group sessions, evaluating the eHealth attitude of trainers, and providing the necessary eHealth training to increase the skills of future e-IMR trainers.

## Appendix

Multimedia Appendix 1 Conceptual framework based on the barriers and incentives for change of different levels of health care, interview items, and data source.

Multimedia Appendix 2 An overview of the eHealth components and implementation of the electronic Illness Management and Recovery (e-IMR) intervention.

Multimedia Appendix 3 Personal characteristics of participants and trainers.

## Abbreviations

e-IMR: electronic Illness Management and Recovery
IM: intervention mapping
IMR: Illness Management and Recovery
IT: information technology
RCT: randomized controlled trial
SMI: severe mental illness
