# Supplementary material for: A Blended Electronic Illness Management and Recovery Program for People With Severe Mental Illness: Qualitative Process Evaluation Alongside a Randomized Controlled Trial
Source: JMIR Ment Health. 2021 Jan 20;8(1):e20860. doi: 10.2196/20860 (PMC7857951; doi:10.2196/20860)
Supplement: Multimedia Appendix 1 [file mental_v8i1e20860_app1.docx]

**Table 1.** Conceptual framework based on the barriers and incentives for change of different levels of health care, interview items, data source.

| Conceptual framework | |  |  | Data source | |
| --- | --- | --- | --- | --- | --- |
| Factors | Determinants | Determinants definitions | Interview items/questions | P | T |
| e-IMR inter-vention | Added value | The extent to which the components of the e-IMR platform had added value to the users and were easy and pleasant to use. | Did you use the eHealth components and how did you estimate the ease or pleasantness of use? | E | E |
|  |  |  | What was your experience in using the eHealth components? Can you describe the added value of the components? | E | E |
|  | Accessibility | The extent to which the e-IMR platform is accessible | Did you access (log-in) the e-IMR platform, at home and in the sessions? What made you couldn’t? | E | E |
|  | Implementation fidelity | The extent to which the implementation of the e-IMR platform is executed as planned | How did you implement the e-IMR platform? What was your experience regarding the implementation? | E | E |
|  | Feasibility | The extent to which the e-IMR platform is practical and fits with current practices | What was your experience in using the eHealth components? How user-friendly was the e-IMR platform? | E | E |
| Partici-pants | Attitude | The perceptions, including preferences, motivations, and self-efficacy that the participants have regarding to using internet, computers, and the e-IMR platform. | What do you think about using the computer related to your health? | B, E | E |
|  | Compliance | The extent to which the participants intend to adhere to using the e-IMR platform | You stopped using the e-IMR platform. What made you do so? | E | E |
|  | Skills & Knowledge | The extent to which the participants have knowledge, expertise, and skills they need to be able to use the e-IMR platform. | Do you have good computer skills; do you need guidance? | B, E | E |
|  |  |  | What was your experience in using the computer? | E | E |
|  | ICT-Resources | The resources that participants have in order to be able to use the e-IMR platform: | Do you have a computer, laptop, smartphone, WIFI, and finances? | B, E | E |
| Partici-pants’ Social context | Social support | The extent to which the participants have support from others in their social context when having difficulties in using the e-IMR platform. | Did you get help at home? What was your experience? | E | E |
|  | Group effect | The extent to which the participants were influenced by other participants in the group in using the e-IMR platform | What did you experience in the group sessions regarding the use of the e-IMR platform? | E | E |
| Trainers | Attitude | The perceptions, including preferences, motivations, and self-efficacy that the trainers have regarding to using internet, computers, and the e-IMR platform. | What do you think about using the computer in the e-IMR intervention and in your contacts with persons with SMI? | E | E |
|  | Skills & Knowledge | The extent to which the trainers have knowledge, expertise, and that they need to be able to use the e-IMR platform. | Do you have e-health experience? |  | B |
|  |  |  | Do you have enough computer skills and knowledge about the e-IMR intervention? |  | E |
| Trainersprofess-sional context | Policy | The extent to which organizational regulations influence the use of the e-IMR platform. | What helped or hindered you in using the e-IMR interventions? | E | E |
|  | ICT-Resources | The resources that organizations have in order to be able to use the e-IMR platform. | What helped or hindered you in using the e-IMR interventions? | E | E |
|  | Work flows | The extent to which trainers are able to adapt their workflow. | What helped or hindered you in using the e-IMR interventions? | E | E |
| General in-depth questions in semi-structured interviews: Can you tell more about why (why not)? What kind of feeling came up? How did you experience ….? What do you think about …..? Can you give an example? | | | | | |
| B: Baseline; E: endpoint; P: Participants; T: Trainers | | | | | |
