# Supplementary material for: A Blended Electronic Illness Management and Recovery Program for People With Severe Mental Illness: Qualitative Process Evaluation Alongside a Randomized Controlled Trial
Source: JMIR Ment Health. 2021 Jan 20;8(1):e20860. doi: 10.2196/20860 (PMC7857951; doi:10.2196/20860)
Supplement: Multimedia Appendix 2 [file mental_v8i1e20860_app2.docx]

**Table 2.** An overview of the eHealth components and implementation of the e-IMR intervention.

| e-IMR components implemented in traditional IMR modules | | | | | eHealth components presence | | | | | |
| --- | --- | --- | --- | --- | --- | --- | --- | --- | --- | --- |
| Modules | | | | | | PTV | GTS | CSS | PSS | SMP |
|  |  | | | e-IMR Welcome page | | X |  |  |  |  |
| 1. | Recovery strategies | | | | | X | X |  |  |  |
| 2. | Practical facts about mental illnesses | | | | | X | X | X | X |  |
| 3. | The stress-vulnerability model | | | | | X | X | X | X | X |
| 4. | Building social support | | | | | X | X | X | X | X |
| 5. | Using medication effectively | | | | | X | X | X | X | X |
| 6. | Drugs and alcohol use | | | | | X | X | X | X | X |
| 7. | Reducing relapses | | | | | X | X | X | X | X |
| 8. | Coping with stress | | | | | X | X | X | X | X |
| 9. | Coping with persistent symptoms | | | | | X | X | X | X |  |
| 10. | Getting your needs met in the mental health system | | | | | X | X | X | X |  |
| 11. | Healthy lifestyles | | | | | X | X | X | X |  |
| Explanation of eHealth components: | | | | | | | | | | |
| e-IMR Welcome page | | | Information about the use of e-IMR and leaded participants to the 11 modules and chapters. Participants could fill in their name after which the platform addressed the participants using their name. The e-IMR chapters of the modules were planned to be used parallel to chapters in the face-to-face sessions. | | | | | | | |
| PTV: Peer Tes-timonial Videos | | | Illustrative videos in every chapter showing peer testimonials to encourage participants to disclose themselves and engage in recovery. The testifying peers were members of the developing group. | | | | | | | |
| GTS: Goal Tracking Sheets | | | Every online chapter automatically reminded the participants to evaluate their personal recovery goals and planned actions. | | | | | | | |
| CSS: Coping Strategies Sheets | | | Introduced after module 1, in every chapter participants were asked to describe successful coping strategies and they were automatically reminded of these strategies in the later chapters. | | | | | | | |
| PSS: Problem-Solving Sheets | | | At the end of each module a problem solving sheet was provided to help to resolve remaining problems within the module, | | | | | | | |
| SMP: Symptom Monitoring Page | | | Introduced after module two (practical facts about mental illnesses) until module nine (coping with persistent symptoms), participants could estimate their burden related to groups of symptoms representing the chapters of module nine: depression, anxiety, hallucinations, delusions, sleep problems, low energy, anger, and concentration problems. Weekly emails with a link to the e-IMR led the participants to the symptom-monitoring page. There symptom estimations were charted in a graph over time. | | | | | | | |
| Workflow | | | At home, or a another place of their convenience, participants enter the e-IMR platform and make the home-work assignments, keep track of their recovery goals, successful coping strategies, work on problem solving sheets, and monitor symptoms. | | | | | | | |
|  |  |  | In the sessions, via a computer and projection, the e-IMR platform was opened to show and discuss the peer testimonials, sheets in which participants completed home work assignments, goal tracking, coping strategies, problem solving, and monitoring. | | | | | | | |
|  |  |  | After finishing a chapter within a module, participants could continue and open the next chapter. They were able to look back to the chapter but could not amend their notes. After closing a module, one of the trainers gave feedback to the participants via the platform and guided the participants to the next module. Participants could not proceed on their own in order to prevent participants are working ahead and loose the parallelism with the group sessions. | | | | | | | |
| Implementation and evaluation of the e-IMR trail | | | | | | | | | | |
| Implementation of the e-IMR | | - Before the start of the trial preparative talks were held with the local IMR-coordinators | | | | | | | | |
|  |  | - In the second IMR session within the intervention group information about the e-IMR was provided to the group members and trainers; and email addresses were collected of the members of the group. All group members were allowed to enter the platform. Participating on the e-IMR was not obliged for participants, because of the known problems with computer availability. | | | | | | | | |
|  |  | - Participants who stated the need for guidance with using a computer and had problems with computer availability were offered help from research assistants with using the website of the e-IMR intervention and finding a place to access a computer. | | | | | | | | |
|  |  | - The trainer-couples were supported in learning how to support participants to use e-IMR, how to install e-IMR on a computer in the session room, and how to use e-IMR during the sessions. One of the trainers was assigned to use the e-IMR platform and communicate with participants via the platform. | | | | | | | | |
|  |  | - Half-way the trial interviews were held with trainers in order to re-introduce the use of e-IMR in the sessions and the participants at home. In reaction to this request, in four out of the seven sites extra e-IMR lessons were organized outside the group sessions. | | | | | | | | |
| Process Evaluation | | An early (explorative) multi center cluster randomized controlled trial was performed between January 2015 and October 2016. At baseline personal characteristics of participants and trainers were gathered. Log-in data were derived from the e-IMR platform. Qualitative data were collected at the endpoint from participants and trainers. Interview items: the e-IMR intervention and implementation, participants, social context of the participants, trainers, and organizational context of the trainers. | | | | | | | | |
