# Supplementary material for: A Blended Electronic Illness Management and Recovery Program for People With Severe Mental Illness: Qualitative Process Evaluation Alongside a Randomized Controlled Trial
Source: JMIR Ment Health. 2021 Jan 20;8(1):e20860. doi: 10.2196/20860 (PMC7857951; doi:10.2196/20860)
Supplement: Multimedia Appendix 3 [file mental_v8i1e20860_app3.docx]

**Table 3. Personal characteristics of participants and trainers.**

|  | | Total group | | | Users | | | | Nonusers | | |
| --- | --- | --- | --- | --- | --- | --- | --- | --- | --- | --- | --- |
| Variables | | n (%) | Mean  (SD) | | n (% within group) | | Mean  (SD) | | n (% within group) | | Mean  (SD) |
| **Participants at baseline** | | 41(100) |  | | 14(34.1) | |  | | 27(65.9) | |  |
| Age | |  | 46.9(11.6) | |  | | 45.2(11) | |  | | 50.14(12.4) |
| Male^*^ | | 11(27) |  | | 1(7) | |  | | 10(37) | |  |
| Diagnoses | |  |  | |  | |  | |  | |  |
| Psychotic disorders | | 14(34) |  | | 4(29) | |  | | 10(38) | |  |
| Mood/anxiety disorders | | 15(37) |  | | 5(36) | |  | | 9(33) | |  |
| Other disorders | | 12(29) |  | | 5(36) | |  | | 8(30) | |  |
| Global assessment of functioning | |  | 50.9(8.2) | |  | | 51.4(8.9) | |  | | 50.5(8) |
| Having a psychiatric comorbidity | | 27(66) |  | | 10(71) | |  | | 17(63) | |  |
| Having a somatic comorbidity | | 23(56) |  | | 9(64) | |  | | 14(51.9) | |  |
| Treatment history | |  |  | |  | |  | |  | |  |
| Years ago since first treatment | |  | 17.2(12) | |  | | 14.4(11) | |  | | 18.6(12.9) |
| Number of admissions | |  | 4.2(4) | |  | | 4.2(3.7) | |  | | 4.1(4.5) |
| Cultural Background | |  |  | |  | |  | |  | |  |
| Dutch | | 37(90) |  | | 14(100) | |  | | 23(85) | |  |
| Turkish, Maroc, Surinam, or English | | 4(10) |  | | 0(0) | |  | | 4(15) | |  |
| In/outpatients | |  |  | |  | |  | |  | |  |
| Independent living | | 30(73) |  | | 12(86) | |  | | 17(63) | |  |
| Supported housing | | 11(27) |  | | 2(14) | |  | | 10(37) | |  |
| Netto income | |  |  | |  | |  | |  | |  |
| ≤ Minimal income | | 31(76) |  | | 11(79) | |  | | 20(74) | |  |
| > Minimal income | | 10(24) |  | | 3(21) | |  | | 7(26) | |  |
| Highest graduated education | |  |  | |  | |  | |  | |  |
| ≤ Middle school | | 26(63) |  | | 9(64) | |  | | 17(63) | |  |
| ≥ High school | | 15(37) |  | | 5(36) | |  | | 10(37) | |  |
| Participant computer resources | |  |  | |  | |  | |  | |  |
| I never use a computer | Agree | 6 (15) |  | | 2 (15) | |  | | 4 (15) | |  |
|  | Disagree | 35 (85) |  | | 12 (85) | |  | | 23 (85) | |  |
| I don’t have a computer | Agree | 8 (20) |  | | 1 (7) | |  | | 7 (26) | |  |
|  | Disagree | 33 (80) |  | | 13 (93) | |  | | 20 (74) | |  |
| Participants’ activity on the e-IMR platform | | |  | |  | |  | |  | |  |
| Logged in on the e-IMR platform | | 23 (56) |  | |  | |  | |  | |  |
| Completed module 1 | | 12 (29) |  | |  | |  | |  | |  |
| Visited monitoring page | | 8 (20) |  | |  | |  | |  | |  |
| **Trainer characteristics at baseline** | | 15(100) |  | |  | |  | |  | |  |
| Age | |  | 46.7(8.8) | |  | |  | |  | |  |
| Male | | 6(40) |  | |  | |  | |  | |  |
| Highest graduated education | |  |  | |  | |  | |  | |  |
| ≤ Middle school | | 8(53) |  | |  | |  | |  | |  |
| ≥ High school | | 7(47) |  | |  | |  | |  | |  |
| Profession | |  |  | |  | |  | |  | |  |
| Peer professional | | 5(33) |  | |  | |  | |  | |  |
| Psychiatric Nurse | | 9(60) |  | |  | |  | |  | |  |
| Social worker | | 1(7) |  | |  | |  | |  | |  |
| Years of experience in mental health | |  | 17.6(11.4) | |  | |  | |  | |  |
| Having experience with eHealth | | 1(7) |  | |  | |  | |  | |  |
| **Participants’ attitude towards computers at baseline** | | | | | | | | | | | |
| Answering options | | strongly disagree | | disagree | | neutral | | agree | | strongly agree | |
| I need guidance with using a computer | | 10 | | 12 | | 5 | | 11 | | 3 | |
| Users | | 5 | | 5 | | 0 | | 3 | | 1 | |
| Non-users | | 5 | | 7 | | 5 | | 8 | | 2 | |
| I have good computer skills | | 6 | | 8 | | 11 | | 11 | | 5 | |
| Users | | 2 | | 5 | | 3 | | 2 | | 2 | |
| Non-users | | 4 | | 3 | | 8 | | 9 | | 3 | |
| n: number; SD: Standard Deviation; ^*^difference between groups of users and non-users is significant (*P*<0,05) | | | | | | | | | | | |
